# Asymmetrical plasmonic absorber and reflector based on tilted Weyl semimetals

**DOI:** 10.1038/s41598-021-94808-y

**Published:** 2021-07-29

**Authors:** Somayeh Oskoui Abdol, Babak Abdollahipour

**Affiliations:** Faculty of physics, University of Tabriz, Tabriz, 51666-16471 Iran

**Keywords:** Optics and photonics, Nanophotonics and plasmonics

## Abstract

We investigate the surface plasmon polariton dispersion and optical spectra of a thin film of tilted Weyl semimetal. Tilted Weyl semimetals possess tilted Weyl cones at the Weyl nodes and are categorized to type-I with closed Fermi surfaces and type-II with overtilted Weyl cones and open Fermi surfaces. We find that the surface plasmon polariton dispersion of this system is nonreciprocal even in the absence of the external magnetic field. Moreover, we demonstrate that the tilt parameter has a profound effect in controlling this nonreciprocity. We reveal that the thin film of type-II Weyl semimetal hosts the surface plasmon polariton modes with the negative group velocity. Furthermore, we show that the angular optical spectra of this structure are highly asymmetric and this angular asymmetry in the absorptivity and reflectivity depends profoundly on the tilt parameter of the tilted Weyl semimetal. These exciting features propose employing the tilted Weyl semimetals in optical sensing devices, optical data storage, and devices for quantum information processing.

## Introduction

Weyl semimetals (WSMs) have recently attracted a surge of research interest due to their massless bulk fermions exhibiting topological properties and offering a great opportunity for applications in the tunable nonreciprocal optical elements^1,2^. WSMs possess linear energy dispersion around the Weyl nodes, where the valence and conduction bands touch each other. To realize a WSM, one of the time-reversal or inversion symmetries should be broken. A WSM with the broken time-reversal symmetry has a pair of Weyl nodes with different chiralities separated in the momentum space that can be regarded as magnetic monopole and anti-monopole in this space^3^. A modified version of WSMs called tilted Weyl semimetals (TWSMs), having tilted Weyl cones, have been realized recently^4–8^. If the tilting velocity exceeds the Fermi velocity the Weyl cones are overtilted with open Fermi surfaces and the related TWSM is categorized as the type-II WSM, otherwise, it will be type-I WSM having closed Fermi surfaces^9–11^. The overtilted Weyl cones in type-II WSMs give rise to a violation of the Lorentz invariance that is manifested in distinct properties of these materials. The tilt of Weyl cones emerges in the number of exotic effects such as quantum transport^12^, non-universal anomalous Hall effect^7^, and squeezed Landau levels^13^.

WSMs and TWSMs are promising materials for application in photonics and plasmonics areas due to their widely tunable chemical potentials and very high electron mobilities. Several studies have been focused on investigating the features of the surface plasmon polaritons (SPPs) in WSMs^14–20^ and TWSMs^21^. Surface plasmons are collective oscillations of free electrons in a conductor that are localized close to its interface with a dielectric and propagate along it. They are accompanied by an electromagnetic field restricted to the interface to realize SPPs. A polarized light impinging on the interface can efficiently excite an SPP mode corresponding to the frequency and the wave vector of the incident light. Its excitation emerges as a peak in the absorptivity or as a dip in the reflectivity spectra of the material. Besides, SPP modes are sensitive to the alteration in the refractive index of the adjacent dielectric media, and this property is extensively utilized for sensing purposes such as biosensing^22^. The exotic and intrinsic features of WSMs and TWSMs that provide the realization of the unidirectional SPP modes without need for the application of the external magnetic field profoundly suggest utilizing these new materials in the sensing devices. Direction-dependent absorption and reflection characteristic of the optical materials is the subject of some recent research papers. An absorber that is capable to absorb light asymmetrically depending on the illumination direction has been introduced based on the structure consisting of a square hole lattice^23^. It has been shown that the left-right asymmetry in the optical reflection can be achieved in a device consisting of an orthorhombic dielectric material that sits atop a 1D grating and is coated with a 3D topological insulator^24^. Moreover, this asymmetry can be enhanced by constructing a periodic multilayer of a topological insulator and an anisotropic dielectric material^25^. It has been demonstrated that evanescent waves engineering can provide a robust platform to control the asymmetric response of metasurfaces for oblique incidence^26,27^.

In this paper, we first investigate features of SPP modes at the surface of a thin film of TWSM, and specifically the influence of the tilt of the Weyl cones on their properties. We show that the tilt parameter can control the nonreciprocity of the SPP modes in the Voigt configuration. This feature reveals a great opportunity for application since we know that the tilt parameter of a TWSM can be effectively tuned via the tension^28^. Furthermore, we apply the Otto configuration to excite these SPP modes and show that in the resulting absorptivity spectrum of the structure dips are generated associated with the attributed SPP modes. In particular, we reveal that the optical spectra of the structure can become highly asymmetric by adjusting the parameters of the system due to the nonreciprocity of the SPP modes. The asymmetric reflection of this system can be utilized for realizing more effective sensing devices.

The remainder of the paper is organized as follows. In “The theoretical model and equations” section we introduce our theoretical model and give the necessary equations for calculating SPPs dispersion relation. The dispersion relation of the SPP modes in a thin film of TWSM is derived in “SPP modes of a thin film of TWSM in the Voigt configuration” section and we present our results for SPP dispersion in this section. The excitation of the SPPs by the method of attenuated total reflection (ATR) is discussed in “Optical spectra of TWSM thin film in the Voigt configuration” section and we study the optical spectra of the structure. Finally, we end by giving a conclusion in “Conclusion” section.

## The theoretical model and equations

The minimal model Hamiltonian for low energy excitations in the vicinity of the tilted Weyl nodes is given by^29^,

1 $$\begin{aligned} H(k)=\hbar {\mathbf {v_{t}}} \cdot {\mathbf {k}} \sigma _0 + \hbar v_{f} \sigma \cdot {\mathbf {k}}, \end{aligned}$$

where $v_{f}$ is Fermi velocity, ${\mathbf {v_{t}}}$ is a vector denoting tilt parameter, ${\mathbf {k}}$ denotes the vector of momentum operator, $\varvec{\sigma }=(\sigma _x, \sigma _y, \sigma _z)$ and $\sigma _0$ indicate Pauli matrices and identity matrix, respectively. Normally, a dimensionless parameter is defined for indicating the tilt of the Weyl cone as $\varvec{\zeta }=v_t/v_f$ that characterizes type of the WSM. $\zeta <1$ denotes the type-I WSMs with tilted Weyl cones and $\zeta >1$ denotes type-II WSMs possessing overtilted Weyl cones. Furthermore, the intrinsic topological nature of WSMs is manifested by the *axion angle* $\theta =2({\mathbf {b}} \cdot {\mathbf {r}}-b_{0} t)$, where ${\mathbf {b}}$ is the vector separating two Weyl nodes with opposite chiralities in momentum space and $b_{0}$ is the separation of them in energy with ${\mathbf {r}}$ and *t* denoting the space and time coordinates, respectively. The modified expression for the displacement field in TWSMs is given by,

2 $$\begin{aligned} D = \varepsilon (\Omega ){\mathbf {E}} +\frac{ic}{\omega }\zeta ^2\nabla \times {\mathbf {B}}+ \frac{i c}{\omega }(\zeta \times \nabla )(\zeta \cdot {\mathbf {B}})+\frac{i e^2}{\pi \hbar \omega c}\dot{\theta }{\mathbf {B}}+\frac{i e^2}{\pi \hbar \omega }(\nabla \theta )\times {\mathbf {E}}, \end{aligned}$$

that has been obtained by minimizing the action field^21^. Here ${\mathbf {E}}$ and ${\mathbf {B}}$ denote the vectors of the electric and magnetic fields, respectively. The diagonal element of the dielectric function, $\varepsilon (\Omega )$, is given by^14^,

3 $$\begin{aligned} \varepsilon (\Omega )=\varepsilon _{0}\left( 1-\dfrac{2r_s g}{3\pi }\dfrac{1}{\Omega ^2}+\dfrac{r_s g}{6\pi }\left[ ln{\dfrac{4{\varepsilon _c}^2}{\vert \Omega ^{2}-4\vert }}+i\pi \Theta (\Omega -2)\right] \right), \end{aligned}$$

where $\Omega =\hbar \omega /E_f$ is the normalized frequency with $E_f$ denoting the Fermi energy, $r_s =\frac{e^2}{\hbar {v_f}{\varepsilon _0}}$ is the effective fine structure constant, $\varepsilon _{0}$ is the background dielectric constant corresponding to the screening inside of the TWSM, *g* is the degeneracy factor, and $\varepsilon _c =E_c/E_f$ denotes the ratio of the cutoff energy to the Fermi energy. Here, we consider the case of broken time reversal symmetry, so that $\dot{\theta }=0$ and $\nabla \theta =2{\mathbf {b}}$. The parameters of TWSM are set as $E_f=0.15\,\hbox {eV}$, $v_f=10^6\,\hbox {m/s}$, $g=24$, $\varepsilon _0=6.2$, $\varepsilon _c=3$, $2b=0.57 \mathring{\mathrm A}^{-1}$^14^.

The electric field attributed to an SPP localized at the interface of a TWSM and a dielectric should decay exponentially into both media. It is considered that the interface of the TWSM with the dielectric medium is located at *x*–*y* plane, thus the electric field takes the following form in both media,

4 $$\begin{aligned} {\mathbf {E}}_j = (E_{j,x},E_{j,y},E_{j,z}){e^{ i{\mathbf{q}}\cdot r_{\bot }}}{e^{ -\kappa _j \left| z \right| }} {e^{ - i\omega t}},\quad j=D, T, \end{aligned}$$

where $r_{\bot }=(x, y)$ and the subscripts *D* and *T* denote the dielectric and TWSM media, respectively. By substituting the electric field (Eq. 4) in the wave equation, a system of linear equations is obtained ($\hat{M }\cdot {\mathbf {E}}=0$). If the direction of SPP propagation be parallel to the *x* axis (${\mathbf {q}} =q\hat{x}$), the direction of ${\mathbf {b}}$ vector should be taken as ${\mathbf {b}} =b\hat{y}$ in the Voigt configuration. However, to have a pronounced effects due to the tilt of Weyl cones we consider that the tilt vector be also parallel to the *x* axis ($\varvec {\zeta } =\zeta \hat{x}$). Hence, the matrix of coefficients for TWSM medium in the Voigt configuration is given by,

5 $$\begin{aligned} \hat{M} = \left( {\begin{array}{*{20}{c}}{-{\kappa _{T}}^2 \lambda ^2 -{k_{0}^2}\varepsilon }&{}{0}&{}{-i q {\kappa _{T}} \lambda ^2-i {k_{0}^2}\varepsilon _b}\\ { 0}&{}{q^2 \lambda ^2- {\kappa _{T}}^2 -{k_{0}^2}\varepsilon }&{}{0}\\ { -i q {\kappa _{T}} \lambda ^2+i {k_{0}^2}\varepsilon _b}&{}{0}&{}{{q}^2\lambda ^2- {k_{0}^2}\varepsilon } \end{array}} \right). \end{aligned}$$

Here we have defined $\lambda ^2=(1-\zeta ^2)$, $\varepsilon _b=\varepsilon _{\infty }(\frac{\omega _b}{\omega })$ with ${\omega _b}= 2{e^2}| b|/\pi \hbar {\varepsilon _{\infty }}$, $\varepsilon _{\infty }=13$, and $k_{0}=\omega /c$ which denotes the wave vector in the vacuum. By setting the determinant of matrix $\hat{M}$ to zero, the following decay constant is obtained for TWSM medium in the Voigt configuration,

6 $$\begin{aligned} \kappa _{T}^{2}= q^2 -{k_{0}^2}\frac{\varepsilon _{v}}{\lambda ^2}~, \quad \varepsilon _{v} = (\varepsilon ^2 -\varepsilon _{b}^2)/\varepsilon, \end{aligned}$$

and for dielectric medium with the dielectric constant $\varepsilon _{d}$, the decay constant is obtained as $\kappa _{D}^{2}= q^2 -k_{0}^2\varepsilon _{d}$. First, we consider the propagation of the electromagnetic waves on the surface of a semi-infinite TWSM with broken time-reversal symmetry in the Voigt configuration. By imposing the boundary condition for tangential components of the electric and magnetic fields, we obtain the SPP dispersion relation for a single interface of TWSM which can be written as,

7 $$\begin{aligned} \varepsilon _d (q^2\lambda ^2-{k_{0}}^2\varepsilon )+\kappa _{D}(\varepsilon \kappa _T-q \varepsilon _b)=0. \end{aligned}$$

**Figure 1:**
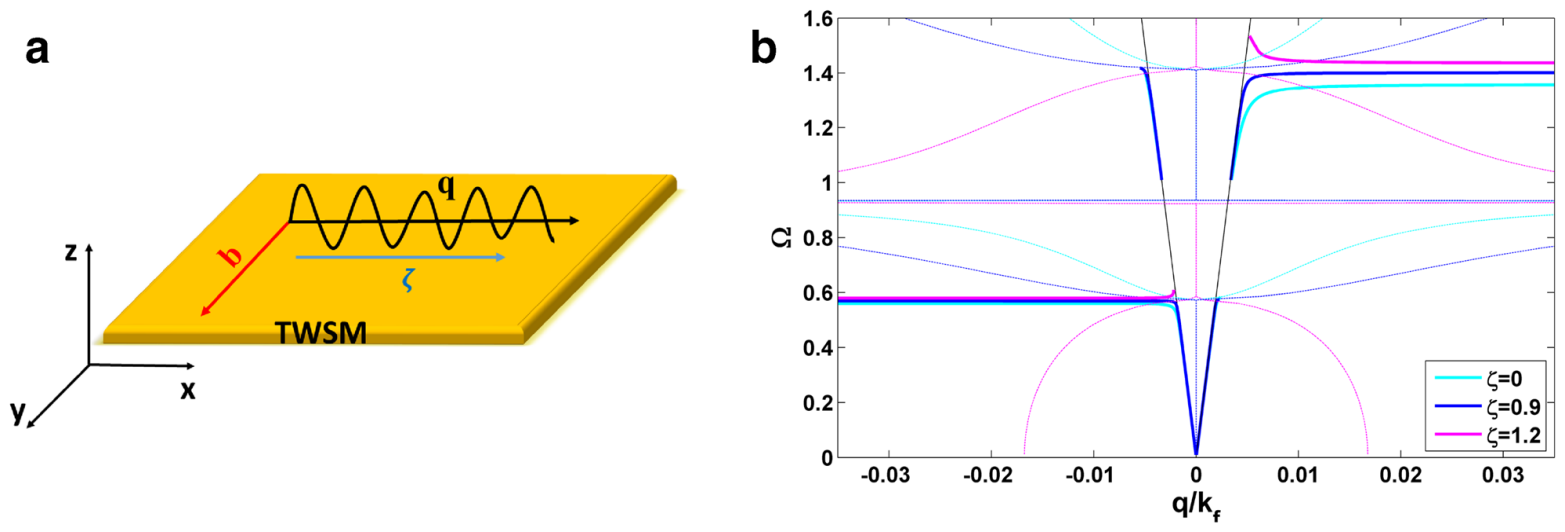
(**a**) Illustration of the Voigt configuration and (**b**) SPP dispersion at the interface of a TWSM and air ($\varepsilon _d=1$) with $E_f=0.15\,\hbox {eV}$, $v_f=10^6\,\hbox {m/s}$, $g=24$, $\varepsilon _0=6.2$, $\varepsilon _c=3$, $2b=0.57 \mathring{\mathrm A}^{-1}$. The thick (thin) curves correspond to SPP (bulk plasmon) dispersion.

This expression for SPP dispersion is distinct from that obtained for the same configuration in Ref.^21^, and reduces to the correct expression of dispersion in the limit of vanishing tilt^15^. We have presented dispersion curves for $\zeta = 0, 0.9, 1.2$ in Fig. 1, where we have plotted $\Omega =\hbar \omega /E_f$ in terms of $q/k_f$ with $k_f=E_f/\hbar v_f$. As we can see, our results don’t display the incorrect feature of down bending of curves in the nonretarded limit indicated in Ref.^21^. Increasing the tilt parameter leads to a shift to higher frequencies in dispersion curves for $q>0$, while there is no considerable change for $q<0$. To emphasize the correctness of the SPP dispersion obtained here, we see that the above dispersion relation is reduced to $\varepsilon _d\lambda ^2 + \varepsilon -\textit{sign}(q)\varepsilon _b=0$ in the nonretarded limit ($q\gg k_0$). This gives rise to the following approximate asymptotic frequencies,

8 $$\begin{aligned} \omega _s\simeq \frac{sign(q)\omega _b+\sqrt{\omega _b^2+ 4\omega _p^2(1+\frac{\varepsilon _d}{\varepsilon _0}\lambda ^2)}}{2(1+\frac{\varepsilon _d}{\varepsilon _0}\lambda ^2)}, \end{aligned}$$

where we have defined $\omega _p=E_f\sqrt{2r_{s}g/3\pi \hbar ^2}$. These asymptotic frequencies coincide with the numerical results plotted in Fig. 1. From the above expression for the asymptotic frequency we can deduce that this system hosts SPP modes when the tilt parameter lies in the range of $0<\zeta <Min\left\{ \sqrt{1+\frac{\varepsilon _0}{\varepsilon _d}}, \sqrt{\frac{\varepsilon _d}{\varepsilon _0}+\frac{\varepsilon _0}{\varepsilon _d}\left( 1+ \frac{\omega _b^2}{\omega _p^2}\right) }\right\}$. Beyond this range of values the SPP modes are not stable.

## SPP modes of a thin film of TWSM in the Voigt configuration

In this section, SPP propagation is investigated at the interfaces of a thin film of TWSM in the Voigt configuration placed between two dielectric layers. By taking into account that only TM mode supports SPP propagation and applying the boundary conditions for tangential components of the electric and magnetic fields at the interfaces of TWSM ($z=\pm a/2$) yields the following relations:

9 $$\begin{aligned} \begin{array}{l} {e^{{\kappa _T}a/2}}{E_{2x}^+ } + {e^{-{\kappa _T}a/2}}{E_{2x}^-} = {E_{1x}},\\ A^{+}{e^{{\kappa _T}a/2}}{E_{2x}^+ } + A^{-}{e^{-{\kappa _T}a/2}}{E_{2x}^-} = ( - \frac{\varepsilon _1}{ \kappa _1}){E_{1x}}, \\ {e^{-{\kappa _T}a/2}}{E_{2x}^+} + {e^{+{\kappa _T}a/2}}{E_{2x}^-} = {E_{3x}}, \\ A^{+}{e^{-{\kappa _T}a/2}}{E_{2x}^+} +A^{-}{e^{+{\kappa _T}a/2}}{E_{2x}^-} = \left( \frac{\varepsilon _{3}}{\kappa _{3}}\right) {E_{3x}}, \end{array} \end{aligned}$$

where $\kappa _{1,3}=q^2-k_{0}^2 \varepsilon _{1,3}$ with $\varepsilon _{1,3}$ denoting the dielectric constants of the dielectric media, $A^{\pm }= \dfrac{\pm \varepsilon \kappa _T+\varepsilon _b q}{q^2\lambda ^2-k_{0}^{2}\varepsilon }$, and $E_{ix}$ with $i=1,2,3$ describes the amplitude of the *x* component of the electric field in different media (see for more detail Refs.^18,19^). Here, we choose these media to correspond to air $\varepsilon _1=1$, TWSM, and water $\varepsilon _3=(1.33)^2$, respectively. By solving this system of equations, the dispersion relation of the SPP modes in the considered structure can be obtained as follows,

10 $$\begin{aligned} \begin{array}{l} \left[ \kappa _1 \kappa _3\varepsilon \varepsilon _v+\varepsilon _b q(\kappa _1\varepsilon _3-\kappa _3\varepsilon _1) +\varepsilon _1\varepsilon _3\gamma ^2 \right] \tanh (a\kappa _{T})+ \varepsilon \kappa _T(\kappa _1\varepsilon _3+\kappa _3\varepsilon _1)=0, \end{array} \end{aligned}$$

where we have defined $\gamma ^2=q^2\lambda ^2-{k_0}^2\varepsilon$. The obtained dispersion relation depends on the orientation of the SPP propagation wave vector (*q*) so that it reflects a nonreciprocal SPP dispersion for this structure. It means that the dispersion relation for positive and negative values of the wave vector is not the same. In the non-retarded limit, $q \gg \kappa _{0}$, Eq. 10 takes the following form,

11 $$\begin{aligned} \varepsilon \varepsilon _v+sign(q)\varepsilon _b(\varepsilon _3-\varepsilon _1)+ \lambda ^2\varepsilon _1\varepsilon _3+\varepsilon (\varepsilon _1+\varepsilon _3)=0. \end{aligned}$$

Using this equation, the asymptotic frequencies for SPP modes of the thin film are approximated as,

12 $$\begin{aligned} \omega _{s1}\simeq \frac{-\omega _b+\sqrt{\omega _b^2+ 4\omega _p^2(1+\frac{\varepsilon _1}{\varepsilon _0}\lambda ^2)}}{2(1+\frac{\varepsilon _1}{\varepsilon _0}\lambda ^2)},\end{aligned}$$

13 $$\begin{aligned} \omega _{s2}\simeq \frac{\omega _b+\sqrt{\omega _b^2+ 4\omega _p^2(1+\frac{\varepsilon _3}{\varepsilon _0}\lambda ^2)}}{2(1+\frac{\varepsilon _3}{\varepsilon _0}\lambda ^2)}. \end{aligned}$$

**Figure 2:**
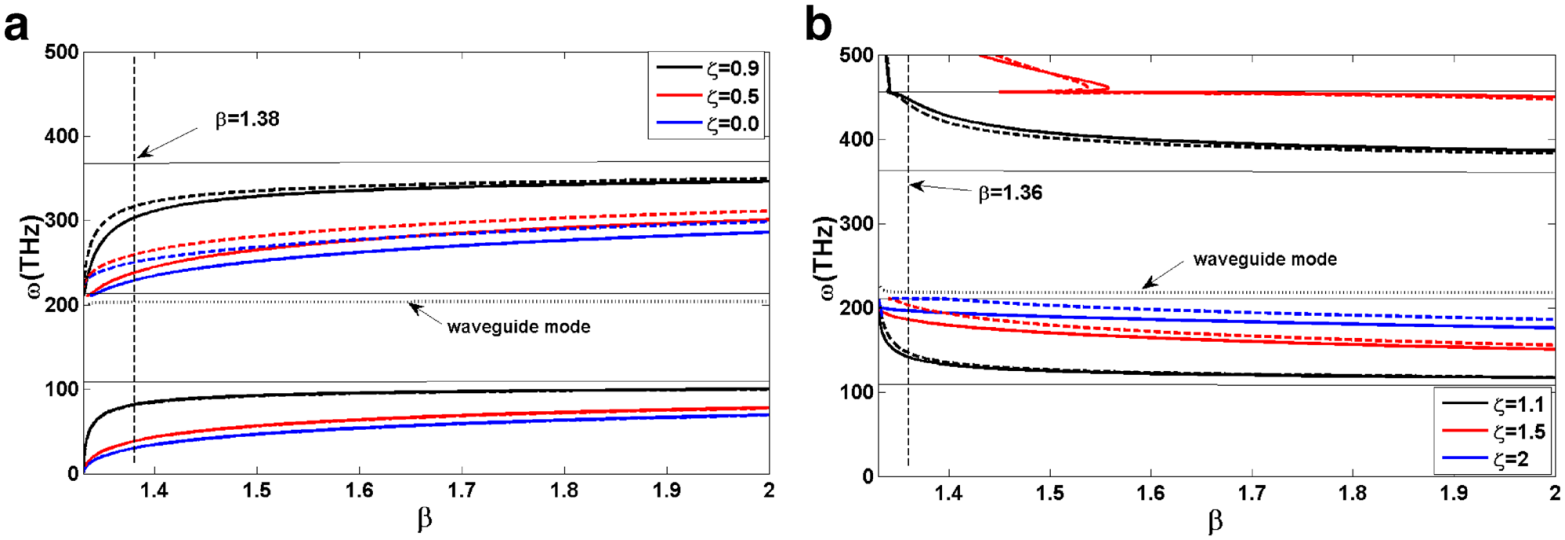
SPP dispersion of TWSM thin film in the Voigt configuration with $E_f=0.15\,\hbox {eV}$, $v_f=10^6\,\hbox {m/s}$, $g=24$, $\varepsilon _0=6.2$, $\varepsilon _c=3$, $2b=0.57 \mathring{\mathrm A}^{-1}$, $a=0.1\,\upmu \hbox {m}$, $\varepsilon _1=1,\varepsilon _3=(1.33)^2$. Panels (**a**), (**b**) correspond to type-I and type-II TWSM for different values of $\zeta$, respectively. The solid lines denote positive wave vector $q>0$ and the dashed lines are for $q<0$. The thin gray curves correspond to bulk plasmon dispersion.

The numerical solution of the dispersion relation given by Eq. 10 results in the dispersion curves for SPP modes in TWSM thin film with Voigt configuration. In order to discuss features of the SPP dispersion we plot it as a function of $\beta =qc/\omega$ for different values of tilt parameter for type-I TWSM ($\zeta =0, 0.5, 0.9$), and for type-II TWSM ($\zeta =1.1, 1.5, 2$) in Fig. 2(a) and (b), respectively. In these figures the solid and dashed lines display the SPP dispersions corresponding to the forward ($q>0$) and backward ($q<0$) propagations. As it is obvious from Fig. 2, the SPP dispersion curves of TWSM thin film are composed of two nonreciprocal bands. Moreover, we find a propagating waveguide mode in addition to the SPP modes. As we can see, this nonreciprocal effect is prominent for the higher (lower) band in type-I (type-II) TWSM. The gap of the dispersion (difference between curves with $q>0$ and $q<0$) decreases for type-I (Fig. 2a) and increases for type-II (Fig. 2b) TWSM by increasing the value of the tilt parameter. For the values of the tilt parameter very close to 1, SPP modes approach the bulk plasmon dispersion indicated by thin gray curves in Fig. 2. Besides, SPP modes in the case of type-II TWSM exhibit the interesting feature of the negative group velocity which is tunable by the tilt parameter. This anomalous dispersion may find potential applications in optical delay lines, optical data storage, and devices for quantum information processing.

**Figure 3:**
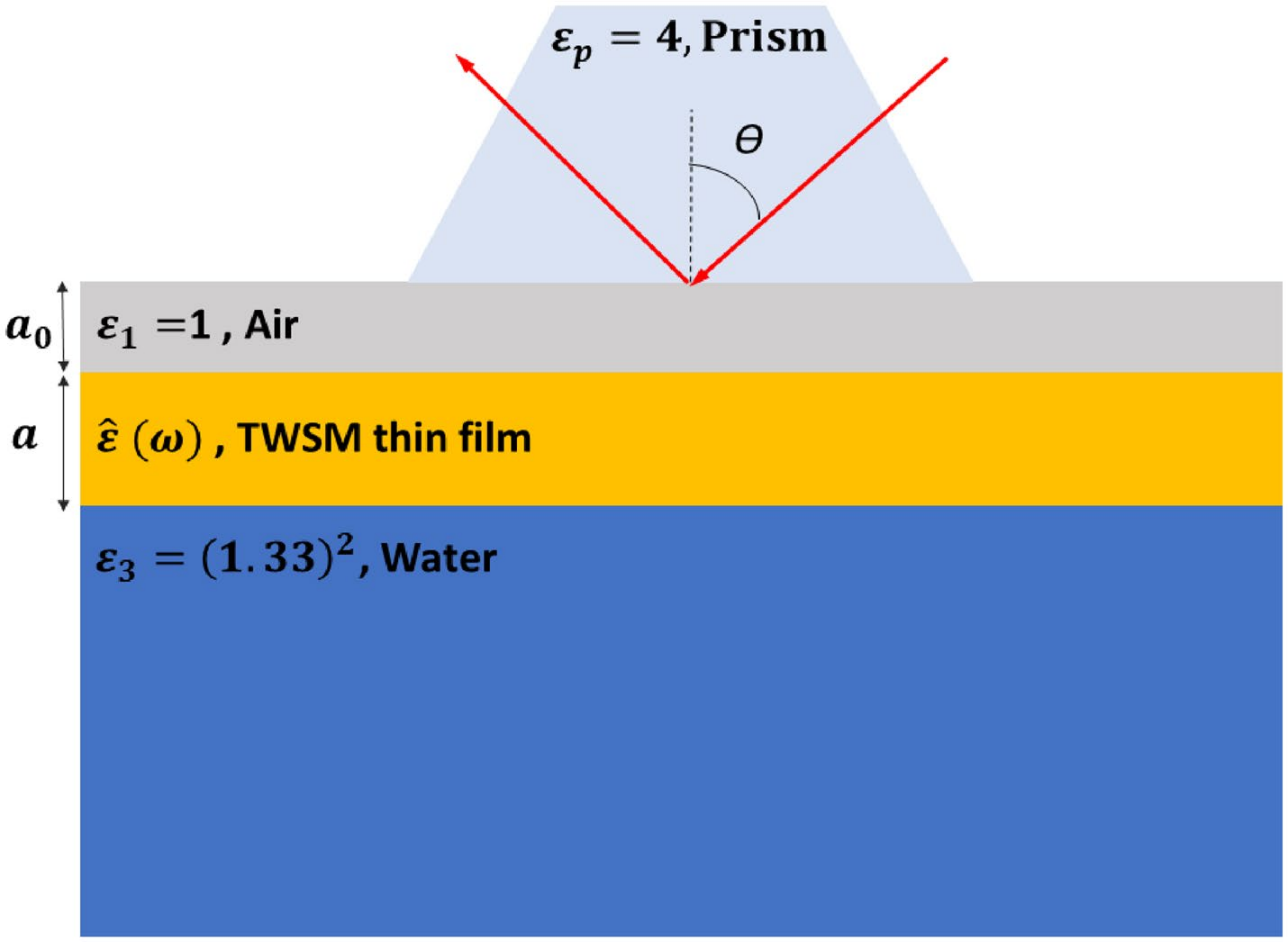
Geometry of ATR experiment.

## Optical spectra of TWSM thin film in the Voigt configuration

To check the dispersion obtained for SPP modes in the previous section, we explore the spectral reflectivity and absorptivity of the thin film of TWSM. SPP modes localized near the interfaces of TWSM can not interact directly with an incoming plane wave due to the momentum mismatch between them. They can be excited by the attenuated total reflection (ATR) method for example with Otto configuration^30^. Therefore, the ATR configuration is used for the calculation of the reflectivity and absorptivity of TWSM thin film which has a complex refractive index. As it has been presented schematically in Fig. 3, the modified ATR structure consists of a prism with a high refractive index and a TWSM film, where the TWSM film is sandwiched between the air and the sensing medium. The permittivity of the prism, air, TWSM film, and sensing medium is denoted by $\varepsilon _p$, $\varepsilon _1$, $\hat{\varepsilon }$ and $\varepsilon _3$, respectively. In the considered structure the thickness of the TWSM film and the air layer is presented by *a*, and $a_0$, respectively. We consider that a laser beam with TM polarization and angular frequency $\omega$ is incident onto the prism/TWSM interface at incidence angle $\theta$. It should be noticed that the angle of the incidence should be large enough so that the inequality of $\sqrt{\varepsilon _p}\sin (\theta )>\sqrt{\varepsilon _3}$ is satisfied to insure happening the total internal reflection.

We utilize the transfer matrix method to investigate the optical spectra of the introduced structure. Thereby, we can write the whole transfer matrix (M) of the structure as the product of the transfer matrices of the constituent layers of it^31^,

14 $$\begin{aligned} \begin{array}{l} M=\left( {\begin{array}{*{20}{c}} {M_{11}}&{}{M_{12}}\\ {M_{21}}&{}{M_{22}} \end{array}} \right) =M_{p}^{-1} M_{air}^{-1} M_{T}^{-1}M_{3}, \end{array} \end{aligned}$$

where the transfer matrices belonging respectively to the prism, air, TWSM, and sensing media can be expressed as,

15 $$\begin{aligned} \begin{array}{l} M_{p}=\left( {\begin{array}{*{20}{c}} {1}&{}{1}\\ {x_p}&{}{-x_p} \end{array}} \right), \\ M_{air}=\left( {\begin{array}{*{20}{c}} {\cos (\kappa _1 a_0)}&{}{\frac{i}{x_1}\sin (\kappa _1 a_0)}\\ {(ix_1)\sin (\kappa _1 a_0)}&{}{\cos (\kappa _1 a_0)} \end{array}} \right), \\ M_{T}=\left( {\begin{array}{*{20}{c}} {\cos (\kappa _T a)+\frac{y_T}{x_T}\sin (\kappa _T a)}&{}{\frac{i}{x_T}\sin (\kappa _T a)}\\ {(ix_T+\frac{iy_T^2}{x_T})\sin (\kappa _T a)}&{}{\cos (\kappa _T a)-\frac{y_T}{x_T}\sin (\kappa _T a)} \end{array}} \right), \\ M_{3}=\left( { \begin{array}{*{20}{c}} {1}\\ { x_3} \end{array}} \right). \end{array} \end{aligned}$$

The parameters employed in Eq. (15) are given by $x_T=\lambda ^2\frac{\varepsilon \kappa _T c}{\omega (\varepsilon ^2-\varepsilon _b^2)}$, $y_T=\lambda ^2\frac{\varepsilon _b qc}{\omega (\varepsilon ^2-\varepsilon _b^2)}$, $x_p=\frac{c \kappa _p}{\omega \varepsilon _p}$, $x_1=\frac{c \kappa _1}{\omega \varepsilon _1}$, and $x_3=\frac{c \kappa _3}{\omega \varepsilon _3}$. In these equations, the transverse wave vector which is conserved throughout the system is given by $q={k_{0}}\sqrt{\varepsilon _p}\sin (\theta )$, and the longitudinal wave vectors in the prism, air, TWSM, and the sensing media respectively are given by,

16 $$\begin{aligned} \begin{array}{l} {\kappa _p={k_0}\sqrt{\varepsilon _p}\cos (\theta )},\\ {\kappa _1={k_0}\sqrt{\varepsilon _1-\varepsilon _p\sin ^2(\theta )}},\\ {\kappa _T={k_0}\sqrt{\varepsilon _v-\varepsilon _p\sin ^2(\theta )}},\\ {\kappa _3={k_0}\sqrt{\varepsilon _3-\varepsilon _p\sin ^2(\theta )}},\\ \end{array} \end{aligned}$$

**Figure 4:**
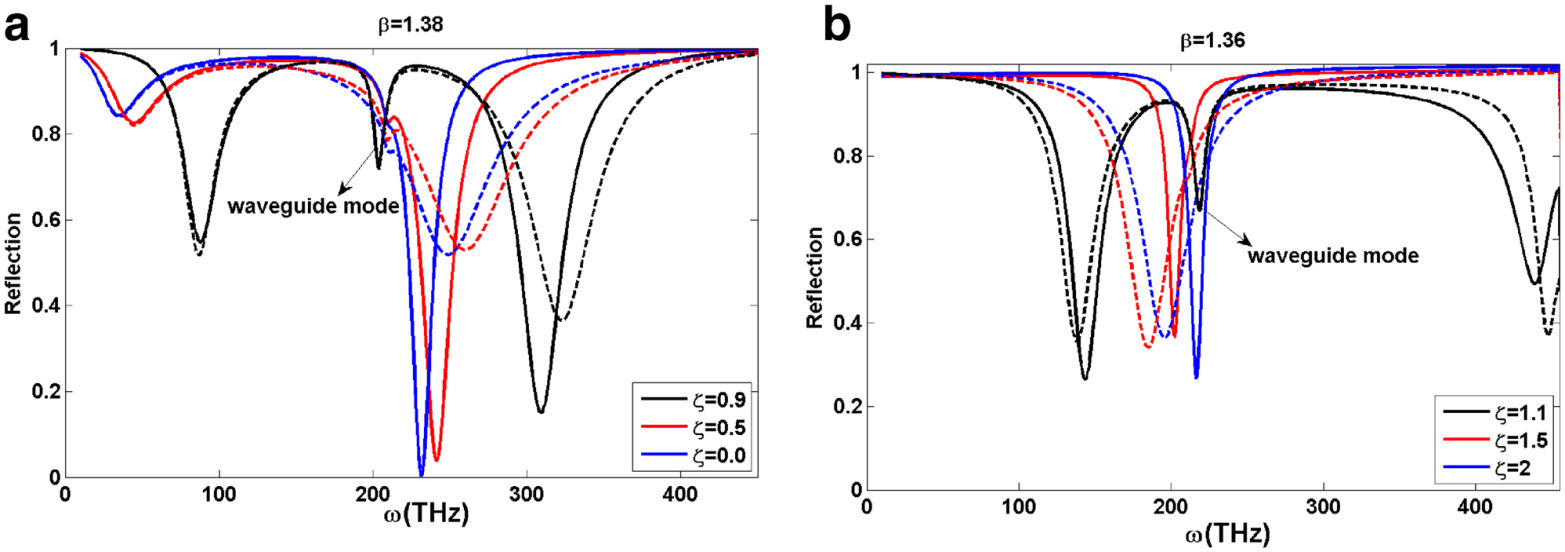
The calculated reflection spectrum of the forward (solid line) and backward (dashed line) in TWSM thin film. Panels (**a**), (**b**) correspond to type-I ($\beta =1.38$) and type-II ($\beta =1.36$) TWSMs for different values of $\zeta$, respectively. Here, we have considered $\varepsilon _p=4$, $\varepsilon _1=1$, $\varepsilon _3=(1.33)^2$, $a_0=a=0.1\,\upmu \hbox {m}$ and the other parameters are the same as Fig. 2.

Eventually, we can get the reflection and transmission coefficients as $r=\dfrac{M_{21}}{M_{11}}$ and $t=\dfrac{1}{M_{11}}$, respectively. Consequently, the reflectivity is given by $R=\vert r \vert ^{2}$, the transmissivity by $T=\vert t \vert ^{2}$, and the absorptivity is determined as $A=1-T-R$. To the numerical calculation of the reflectivity and absorptivity of the TWSM thin film, we introduce a damping parameter via the replacing of the frequency by $\omega \rightarrow \omega +i \tau$. Throughout our calculation, we consider the damping constant to be a small value given by $\tau = 0.02\omega$. The TM polarized ATR reflection spectrums for the type-I and type-II TWSM thin films with $\varepsilon _p=4$, $\varepsilon _1=1$, $\varepsilon _3=(1.33)^2$, $a_0=a=0.1\,\upmu \hbox {m}$ and specific values of $\beta$ are plotted in Fig. 4. In these figures, the solid and dashed lines represent the reflection spectrums for the forward ($q>0$) and backward ($q<0$) propagations. As we can see from the figures, there are dips in the ATR spectrum at frequencies that correspond exactly with the frequencies of SPP modes presented in Fig. 2 for both type-I and type-II cases of TWSM thin films. Existence of these peaks at the ATR spectrum reveals the excitation of the SPP modes in the considered structure. Although, there is an extra dip in the reflection spectra for the tilt parameters close to one in both type-I and type-II TWSMs. Comparison with Fig. 2 shows that we can attribute these dips to the waveguide modes. Furthermore, we observe that in both cases of type-I and type-II TWSM thin films the frequencies where dips happen shift to the higher frequencies in complete accordance with the dispersion curves represented in Fig. 2.

**Figure 5:**
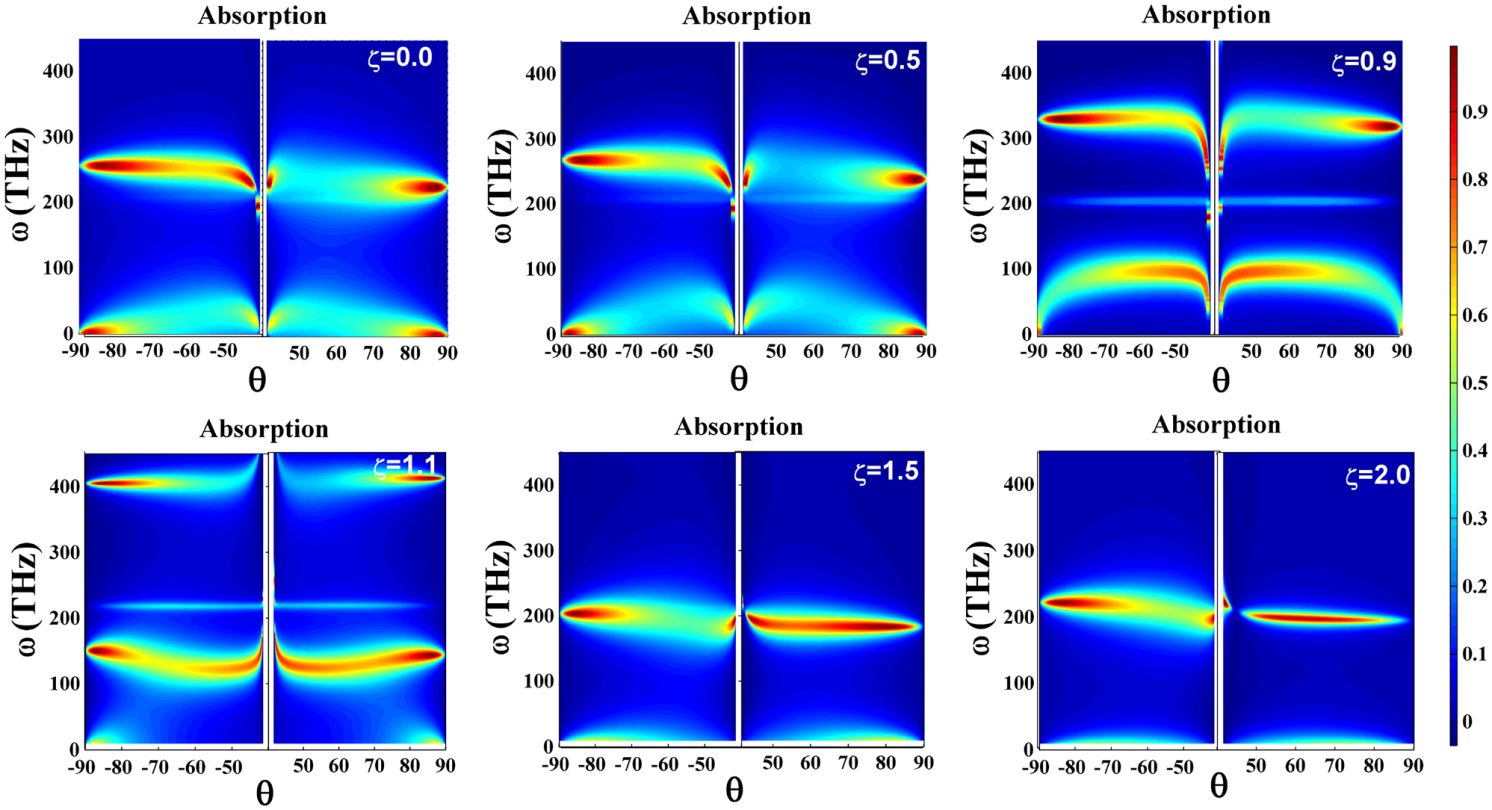
Absorption spectrum (A) of TM-plarized light incident on TWSM thin film in the Voigt configuration with the thickness $a=0.1\,\upmu \hbox {m}$ surrounded by the air $\varepsilon _1=1$ and sensing media $\varepsilon _3=(1.33)^2$ in terms of frequency and incident angle for different values of tilt parameter $\zeta =0.0, 0.5, 0.9, 1.1, 1.5, 2$. The other parameters are the same as Fig. 4.

Now, let us discuss dependence of the optical spectra of TWSM thin film on the angle of incidence. In Fig. 5 we have shown the density plots of the absorption in terms of the frequency and both positive and negative angles of incidence for different values of the tilt parameter. These density plots reveal the SPP modes as regions with high values of absorption. However, we observe new features which were not clear from the SPP dispersion. Comparison of the plots belonging to the positive and negative values of the incident angle clearly shows that there is an asymmetry in the absorption and hence in the reflection spectra of the structure. Furthermore, we see that variation of the tilt parameter profoundly affect the asymmetry in the optical spectra of TWSM thin film. In the case of type-I TWSM ($\zeta <1$) asymmetry in the reflection is only observed in the upper SPP modes and the lower modes are symmetric. However, for type-II TWSM ($\zeta >1$) angular asymmetry in the absorption and reflection spectra also appears in the lower band and, as it is obvious from the figures, enhances by increasing the tilt parameter. Moreover, we find that in the case of type-I TWSM variation of the absorption intensity by increasing the angle from $\theta =\sin ^{-1}(\sqrt{\varepsilon _3}/\sqrt{\varepsilon _p})\simeq 41.7^{\circ }$ to $\theta =90^{\circ }$ is more considerable when SPP propagation is along the tilt direction (positive angles) than the reverse one. While, in type-II TWSM considerable variation happens for SPP modes propagating in the reverse direction of tilt (negative angles).

**Figure 6:**
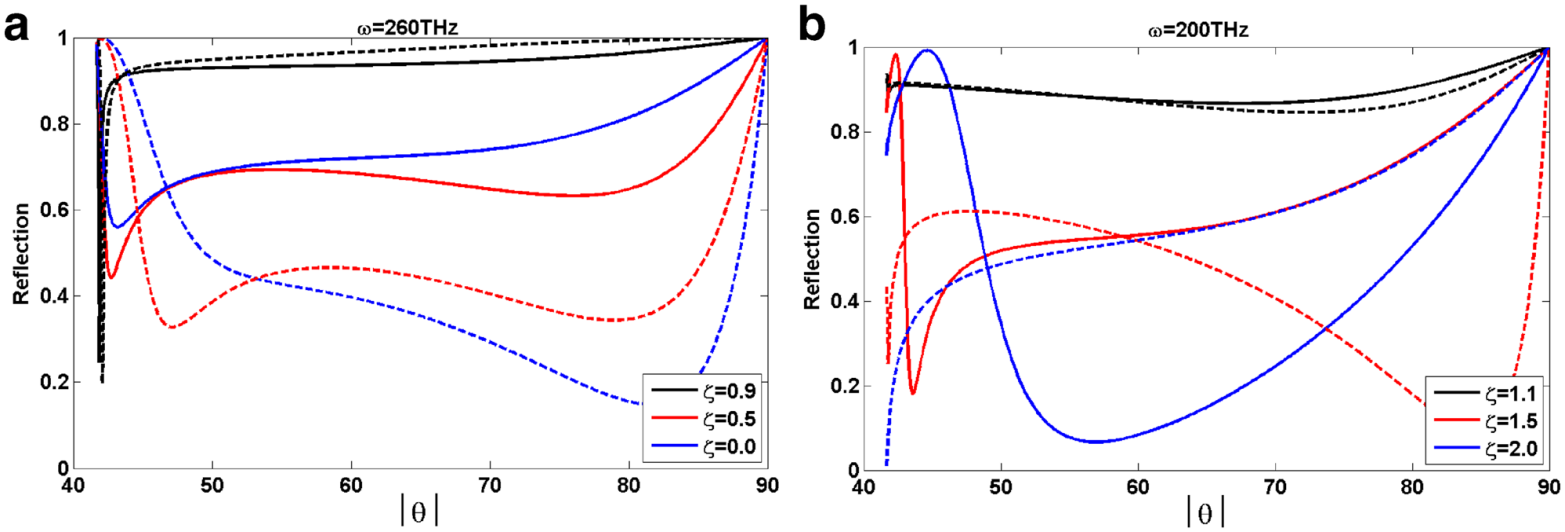
Reflection spectrum (R) of TM-polarized light incident on TWSM thin film in the Voigt configuration with the thickness of $a=0.1\,\upmu \hbox {m}$ surrounded by the air $\varepsilon _1=1$ and sensing media $\varepsilon _3=(1.33)^2$ in terms of the incident angle for different values of the tilt parameter. Panels (**a**), (**b**) correspond to type-I and type-II TWSMs with frequencies $\omega =260$THz and $\omega =200$THz, respectively. The other parameters are the same as Fig. 4.

We have plotted in Fig. 6 the reflection spectrum of TWSM thin film in terms of the angle of incidence to emphasize asymmetry of the reflection spectrum and clear observation of its angular variation. In this figure, the solid lines represent reflection spectrum for positive angles (forward) and the dashed lines are for negative one (backward). By comparing the reflection spectrum for positive and negative angles we found that in type-I TWSM the angular asymmetry suppresses by increasing the tilt parameter and vanishes completely in the extreme case of $\zeta =1$, while it enhances by increasing the tilt parameter in the case of type-II TWSM. Besides, we observe that the angle with largest asymmetry and the angles where maximum and minimum of the reflection happen depend on the tilt parameter. Therefore, we can conclude that the asymmetric reflection and absorption spectra of the TWSM thin film is controllable by its tilt parameter.

## Conclusion

In summary, we have investigated SPP dispersion in a thin film of TWSM where the Weyl cones at the touching points of valence and conduction bands are tilted. The effects of tilted energy spectrum on SPP dispersion and ATR spectra of this structure were analyzed in detail. We found that SPP modes in this structure are nonreciprocal in the Voigt configuration in the absence of the external magnetic field and more importantly, this nonreciprocity is tunable by the tilt parameter. Moreover, we showed that SPP modes in the case of type-II tilted Weyl semimetal exhibit a negative group velocity. These tunable nonreciprocal SPP modes are promising for the design of unidirectional optical devices. Furthermore, it has been revealed that ATR spectra of the thin film of TWSM are profoundly sensitive to the direction and amplitude of the tilt. Besides, we showed that this structure possesses an asymmetric angular absorptivity and hence reflectivity. This asymmetric reflection spectrum is more pronounced in the case of type-II TWSM and it enhances by increasing the tilt parameter such that at some angles nearly perfect asymmetry can be observed. This asymmetric optical spectra can be used for more sensitive sensing purposes.
